# Breast adenoid cystic carcinoma: a report of seven cases and literature review

**DOI:** 10.1186/s12893-022-01560-9

**Published:** 2022-03-24

**Authors:** Meilin Zhang, Yanbiao Liu, Hongguang Yang, Feng Jin, Ang Zheng

**Affiliations:** 1 Department of Breast Surgery, the First Affiliated Hospital of China Medical University, Shenyang, Liaoning China; 2 Department of Burn Plastic Surgery, Chaoyang Central Hospital, Chaoyang, Liaoning China

**Keywords:** Breast, Adenoid cystic carcinoma, Treatment, Prognosis, Case report

## Abstract

**Background:**

Primary adenoid cystic carcinoma (ACC) of breast is rarely seen clinically. It is a special subtype of triple-negative breast cancer characterized by low expression of Ki-67, low malignant potential, slow progression and favorable prognosis. To date, treatment for this disease is controversial and no consensus is reached. We analyzed clinical manifestations and pathological characteristics of seven primary breast ACC cases and reported in combination with literature review to promote understanding, diagnosis and treatment of this disease.

**Case presentation:**

We collected seven breast ACC cases pathologically diagnosed and treated in Department of breast surgery of the First Affiliated Hospital of China Medical University from January 2015 to December 2018. We organized and summarized the clinical, imaging, pathological and prognostic information and performed statistical analysis. The median age was 60 years (ranging from 54 to 64 years). Tumors of all patients were detected by immunohistochemistry. Molecular types were mostly triple negative (4/7), and Ki-67 expression was low (5/7). Lymph node metastases were absent in all patients received axillary lymph node surgery. Median follow-up time was 39 months (ranging from 25 to 68 months). There was no occurrence of relapse, distant metastasis or death.

**Conclusion:**

Breast ACC is accompanied with favorable diagnosis, which is different from typical triple-negative breast cancer. Accurate diagnosis of ACC is particularly important.

## Background

Adenoid cystic carcinoma (ACC) is a rare type of breast cancer with the incidence of less than 0.1%. As reported, 5-year, 10-year and 15-year relative survival rates of this disease are 98.1%, 94.9% and 91.4% respectively [1]. It occurs mostly in female patients and rarely in male patients [2]. Morphologically, ACC is presented as a mixture composed of tubular-trabecular, cribriform and solid structures in different proportions. That’s also the reason why it is easy to be misdiagnosed clinically. Breast ACC is a special subtype of triple-negative breast cancer (TNBC) with low expression of Ki-67, favorable prognosis and rare axillary lymph node metastasis. Local recurrence and distant metastasis are relatively common within ten years, with lung as the most commonly metastasized organ [3]. In 2017, Mhamdi et al. reported a 65-year-old woman diagnosed with breast ACC with lung, kidney and brain metastases [4]. It is necessary to make a detailed clinical and pathological analysis of breast ACC in view of its complex histological morphology.

## Case presentation

Seven cases pathologically diagnosed as breast ACC and received treatment in Department of breast surgery of the First Affiliated Hospital of China Medical University from January 2015 to December 2018 were reviewed. The median age was 60 years, ranging from 54 to 64 years. All patients were postmenopausal. Only one patient claimed the family history of breast cancer. Initial symptoms of these patients were all palpable breast masses, and only two of them complained of pain. Four patients were with left-sided neoplasm and three were with right-sided neoplasm. Tumors were located in superior-lateral quadrant in five cases, superior-medial quadrant in one case, and inferior-medial quadrant in one case. Four patients had tumor larger than 2 cm and three patients had tumor smaller than 2 cm in diameter. In addition, seven patients in this study underwent mammography and breast ultrasound. However, no distinctive features were found in in both imaging techniques. According to BIRADS classification system, there were three cases in 4C category, one case in five category, two cases in 4B category and one case in 4A category respectively. No enlarged lymph node was found by clinical and imaging evaluation. All of the patients were diagnosed as pure ACC by routine paraffin pathology. Immunohistochemical examination on pathological sections revealed that seven patients were negative for the expression of human epidermal growth factor receptor 2 (HER2) and only three of them were positive for the expression of hormone receptors. Four patients underwent modified radical mastectomy (MRM), one patient underwent mastectomy with sentinel lymph node biopsy (SLNB), one patient underwent breast conservation surgery (BCS) with SLNB and one patient underwent lumpectomy alone. Six patients undergoing axillary lymph node surgery did not develop axillary lymph node metastasis. One patient did not receive any adjuvant treatment after operation. Four patients received adjuvant chemotherapy (CT), and one of them received subsequent radiotherapy (RT) within half a year. Two patients received endocrine therapy (Table 1). Median follow-up time was 39 months, ranging from 25 to 68 months. Time of the last follow-up was December 2020. No recurrence, metastasis or mortality occurred in seven patients during the follow-up period.

**Table 1:** Clinical characteristics and pathological features

Case number	Age	Site	Tumor size (cm)	Tenderness	TNM stage	Surgery	Axillary management	Axillary lymph node mets	ER/PR/HER2	Ki67	Treatment
1	60	UOQ	≥ 2	Yes	II	MRM	ALND	No	−*/* +/−	15%	HT
2	54	UOQ	< 2	No	I	MRM	ALND	No	−*/*−*/*−	30%	CT
3	64	UOQ	< 2	No	I	MRM	ALND	No	+ */* +/−	5%	CT
4	60	UIQ	≥ 2	Yes	II	Lumpectomy	–	–	−*/*−*/*−	20%	No
5	55	UOQ	≥ 2	No	II	BCS	SLNB	No	−*/*−*/*−	20%	CT + RT
6	62	LIQ	≥ 2	No	II	MRM	ALND	No	−*/*−*/*−	80%	CT
7	64	UOQ	< 2	No	I	MRM	SLNB	No	+/−*/*−	5%	HT

*UOQ* upper outer quadrant, *UIQ* upper inner quadrant, *LIQ* lower inner quadrant, *MRM* modified radical mastectomy, *BCS* breast conservation surgery, *ALND* axillary lymph node dissection, *SLNB* sentinel lymph node biopsy, *HT* hormone therapy, *CT* chemotherapy, *RT* radiotherapy

## Discussion

### Clinical manifestation

There have been reports on ACC in salivary glands, digestive tract, external auditory canal, skin, uterus, and breast cancer [5, 6]. It is reported that ACC mostly occurs in superior lateral quadrant or below areola of breast. Patients are mostly admitted to hospital with the chief complaint of palpable masses. In most cases, the mass is in solitary and cases of multiple masses are rarely reported [7]. Seven patients in this study were reported with single tumor, and all the tumors of five patients were located in superior lateral quadrant. In addition, SIMONA et al. recommend a combination of LOCalizer™ and Intraoperative Ultrasound for localization and surgery in patients with non-palpable breast masses. The dual technique provides not only accurate localization but also better oncology and cosmetic results. Importantly, it also gives effective treatment to patients with non-palpable breast lesions [8]. As reported, pain in the affected area is another characteristic symptom of this disease, accounting for about 14% of all patients. Kashiwagi et al. revealed that such pain was related to perineural infiltration of tumor cells and contraction of myoepithelial cells [9]. Interestingly, most patients felt no pain at the first time of seeing a doctor. In our study, only two patients complained of occasional pain. It was reported that the average diameter of such tumors was 2–3 cm, and the maximum diameter ever seen was 15 cm [10].

### Imaging manifestation

There is no significant specificity in the imaging presentation of primary breast ACC based on imaging of previous cases. It has been reported that the X-ray appearance of breast ACC can be irregular and high-density mass with fuzzy edge, containing slightly low-density or lipoid density lesions, with less calcification. Ultrasound appearance of breast ACC is non-mass like lesion with high echo and no distribution along direction of catheters. Likewise, there are some valuable findings on MRI. Most lesions of breast ACC are clear. On T2WI, large breast ACC can show extensive internal septum of high and low signal, which can be enhanced in delayed phase [11, 12]. Therefore, Katrina et al. concluded that combination of multiple imaging examinations could increase diagnostic efficiency, despite final diagnosis still depended on pathology [13].

### Pathological grading and features

#### Pathological grading

The pathological grading of ACC is disputed. According to the cell structure, tumors composed of tubular or cribriform structures alone are classified into histological grade I; those with solid component < 30% are classified into histological grade II and those with solid component ≥ 30% are classified into histological grade III. The higher the percentage of solid components, the worse the prognosis of the patients [14]. Foschini et al. put forward another new classification standard of breast ACC: Grade I is the classic type ACC with tubular and cribriform features, which possesses favorable prognosis, meaning rare recurrence or metastasis after surgical. Grade II corresponds to solid breast adenoid cystic carcinoma (SBACC) with basal like features. Axillary lymph node metastases and local recurrence in this type are common, but the prognosis seems to be well. Grade III corresponds to the area of ACC malignant transformation, which may lead to distant metastasis and death [15]. There is also another classification system: classic ACC is classified into low grade and solid adenoid cystic carcinoma with basal cell like features is classified into high grade [3]. In a word, although there are debates on the grading system of breast ACC, we insist that it is crucial to grade this tumor appropriately, in terms of its potential impact on clinical treatment.

#### Pathological features

**Fig. 1:**
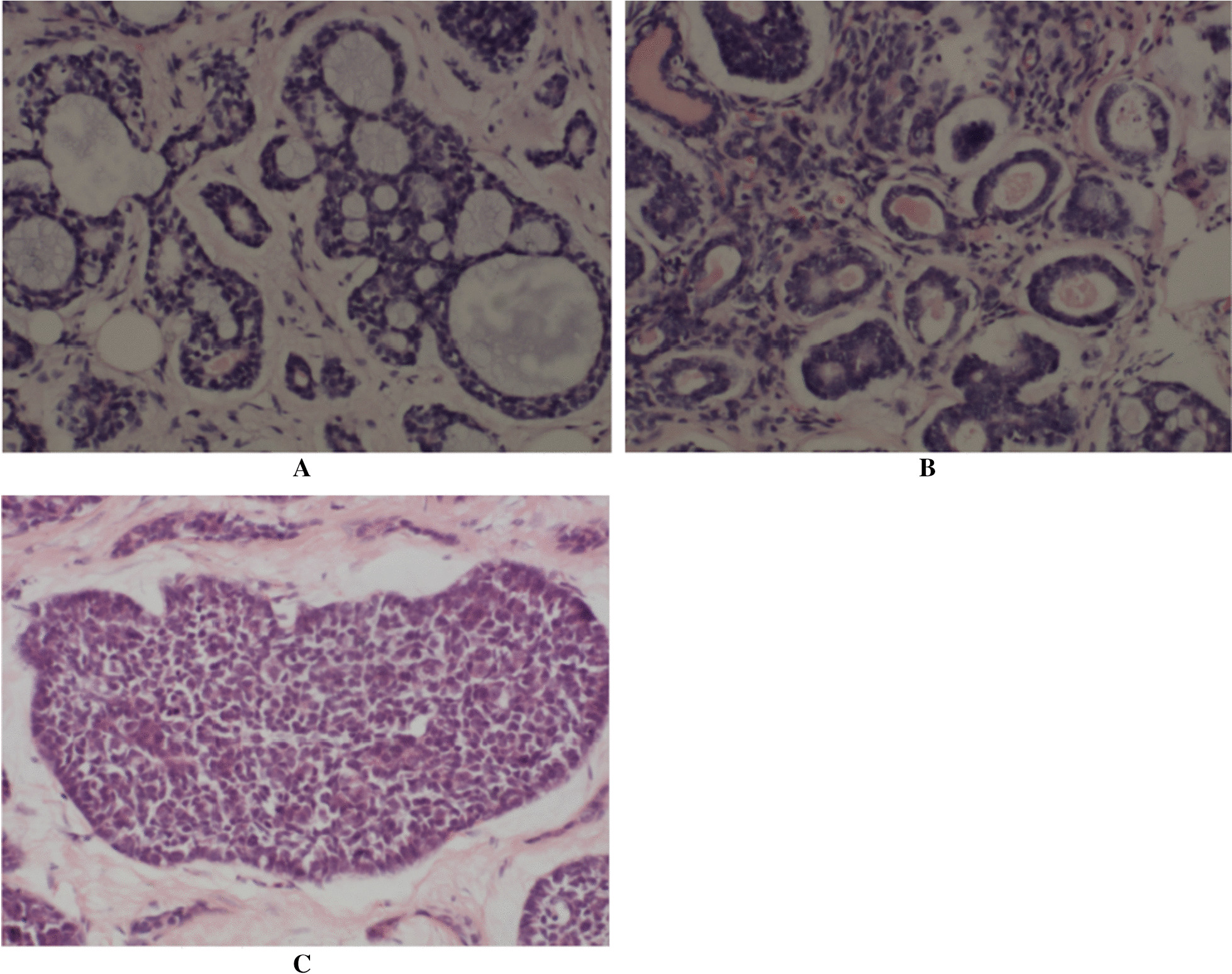
Three histological subtypes of breast ACC (**A**, **B**, **C**). Cribriform (**A**). Tubular-trabecular (**B**). Solid (**C**). HE, ×400

Under the light microscope, three common configurations can be seen: cribriform, tubular-trabecular and solid (Fig. 1). These structures are often mixed, exhibiting cystic and glandular changes or solid lamellar arrangement. The tumor is mainly composed of three kinds of cells: glandular epithelium cells, basal like cells and myoepithelial cells. Moreover, we can observe squamous cell metaplasia and sebaceous cell differentiation.

### Genetic alteration

MYB is the first discovered proto-oncogene located in 6q22-23, which has strong carcinogenic effect and is known to be expressed in a variety of malignant tumors. Nuclear factor IB (NFIB) is a member of the NFI family and serves as a protein coding gene located in 9p23-24. It plays an important role in cell proliferation, apoptosis and development. ACC repetitive translocation t (6; 9) (q22-23; p23-24) leads to the fusion of MYB and NFIB, which is the main molecular mechanism of the disease. A large number of studies have shown that the fusion of MYB and NFIB is closely related to the occurrence and development of breast ACC. This fusion gene has no correlation with the location of ACC, meaning it can be detected in both primary and metastatic lesions. While reports of this fusion gene in other disease are rare, signifying its high specificity in ACC. Some studies have revealed that high expression of MYB protein can be detected even with negative expression of the fusion gene. Based on these findings, it was speculated that MYB played a vital role in ACC while NFIB just assisted fusion of proteins [16, 17]. It has been reported that detection rate of MYB-NFIB fusion gene in breast ACC is discrepant, related to tumor treatment therapy and detection methods. Brill et al. found that detection rate of the fusion gene in frozen samples was higher than that in paraffin preserved samples [16]. Some studies have also shown that MYBs labeled by immunohistochemistry (IHC) has higher sensitivity and specificity than those labeled by fluorescence in situ hybridization (FISH) [18]. Wetterskog et al. reported a detection rate of 92.3% by FISH and 30.8% by Reverse Transcription-Polymerase Chain Reaction [19]. Therefore, selecting an appropriate detection method can improve the detection rate. MYB-NFIB fusion gene and MYB gene play a key role in the molecular pathogenesis of ACC and are expected to be therapeutic targets. We still face many unknown challenges along the way.

### Differential diagnosis

About 50% of breast ACCs are misdiagnosed [1]. In order to avoid incorrect classification, it is necessary to use strict diagnostic criteria, and it is particularly important to get the exact pathological diagnosis before making the treatment plan systematically. Some other diseases that can be differentially diagnosed are as follows:

#### Collagen corpuscle disease

Collagen corpuscle disease is a kind of pathological change under the microscope. While ACC is an independent invasive disease with visible tumors. There are some similarities between these two diseases in morphology of the sieve corpuscles. Both of them have substances like basement membrane. While corpuscles of collagen corpuscle disease have characteristic filiform, radial and coil like shapes and those of ACC contain interstitial or mucinous components, lacking structural characteristics.

#### Invasive cribriform carcinoma

Similar to ACC, invasive cribriform carcinoma possesses an obvious cribriform structure. But its cribriform cell nest is more irregular. The cribriform lining cells of invasive cribriform carcinoma lack expression of basal like cells and myoepithelium. The wedge-shaped pores do not contain matrix components, but rather protein mucus secretions and necrotic tissue. There is no eosinophilic basement membrane like substances around the cell nest. And the expression of ER and PR is often positive while smooth muscle actin and P63 are usually negative.

#### Cribriform ductal carcinoma in situ

Cribriform ductal carcinoma in situ is intraductal. The tumor cells show the features of glandular epithelium. ER and PR are often diffusely positive in cribriform ductal carcinoma in situ while they are often negative in ACC. There are no basal like cells and myoepithelium in the glandular cavity. The contents in the lumen are different from those in the pseudo-lumen of ACC.

#### Adenoid invasive ductal carcinoma

Cellular pleomorphism and atypia are more pronounced in adenoid invasive ductal carcinoma. There are more prominent vesicular nucleus and more abundant cytoplasm compared with ACC. Also, the former lack myoepithelium and obvious extracellular mucus.

### Treatment and prognosis

#### Surgical treatment

Surgery is now recognized as the primary treatment for breast ACC patients. However, due to the rarity of this pathological type, there is no clear guidence in the selection of detailed surgical method for this disease, resulting in differences in treatment. Ro et al. suggested that the operation method should be selected according to the ACC grade. Tumor lumpectomy should be used for grade I tumors, mastectomy should be used for grade II tumors, and mastectomy plus lymph node dissection should be used for grade III tumors [20]. Here, we will discuss the operation methods of classic breast ACC and SBACC separately.

For classic breast ACC, it has been reported that relapse occurred after local excision by a clinical study including 478 cases of breast ACC patients [21]. The treatment includes tumor lumpectomy plus adjuvant RT, tumor lumpectomy alone, mastectomy alone and mastectomy plus adjuvant RT. Through Kaplan–Meier analysis, patients receiving tumor lumpectomy plus adjuvant RT had better survival compared with other patients, indicating lumpectomy plus adjuvant RT can improve the survival and BCS is a reasonable choice for breast ACC patients [22].

The axillary lymph node metastasis of classic breast ACC is rare, generally 0–2%. Axillary lymph node dissection (ALND) is not necessary if there is no preoperative definite clinical evidence of axillary lymph node metastasis. Thompson et al. investigated 244 patients with confirmed breast ACC, discovering that patients with unknown lymph node status have the same favorable 10-year relative cumulative survival rate as known non-lymph node metastasis patients. Therefore, the author believed that ALND was not necessary for patients with breast ACC, especially for patients with T1 stage [23]. Kulkarni et al. designed a clinical study and included 933 patients with breast ACC, among whom 6% received axillary lymph node assessment and only 5% were axillary lymph node positive. He came to the same conclusion that ALND was not necessary for breast ACC patients [24]. However, when patients are with other tumors, or in the case of high-grade lesions, and diameter of breast tumor is larger than 3 cm, SLNB is a wise choice [3, 10]. At present, ALND is not recommended for classic breast ACC.

For SBACC, Shin et al. studied nine patients with SBACC and discovered that treatment of SBACC was different from that of traditional breast ACC. In six patients undergoing ALND, two of them were with axillary lymph node metastasis. Researchers have suggested that if there was no clinical evidence of obvious axillary lymph node metastasis, SLNB or low lymph node dissection should be carried out first. If axillary lymph node metastasis occurred, postoperative adjuvant CT would be non-avoidable. Therefore, SBACC is more invasive and has stronger axillary lymph node metastasis ability than classic ACC. And it seems to have a better prognosis than invasive ductal carcinoma in the same stage [25]. However, there are not immutable therapy regimens. Individual treatments according to specific circumstances of patients are the eternal truth.

#### Seroma formation

MRM is one of the common operations in breast surgery. Breast cancer patients who receive ALND have postoperative complications such as lymphedema, postoperative bleeding, seroma formation, skin paresthesia and upper limb dysfunction. Among them, seroma formation cannot be ignored, the incidence of 3%-85% [26]. Chronic seroma may lead to infection, overloading of the affected upper limbs, and lymphedema. In clinic, how to reduce the incidence of postoperative seroma has important clinical significance for improving the prognosis of breast cancer patients. Claudio et al. analyzed 100 patients with locally advanced breast cancer who underwent ALND and divided the patients into four groups according to the device utilized during the operation: Electrocautery, Harmonic Scalpel, LigaSure and Thunderbeat. The results found that the use of Thunderbeat could significantly reduce seroma formation, intraoperative blood loss and postoperative drainage. As you can see, the use of advanced hemostasis devices is highly advisable when performing ALND [27]. In addition, fibrin glue has also received attention in seroma formation. Giovanni et al. enrolled 30 elderly breast cancer patients who underwent ALND. Although they believed that fibrin glue could not prevent the formation of seroma, it could reduce the seroma extent, duration and length of hospital stay of the patients, thereby improving the prognosis of the patients [28]. Moreover, some clinical factors have been confirmed to be related to the seroma formation and can effectively reduce the occurrence of seroma, such as reduction of dead space [29, 30], suction drainage [31] and use of octreotide [32, 33]. Hypertension, diabetes mellitus and a high body mass index have been confirmed as risk factors for seroma formation by studies [34, 35]. Therefore, it is essential to identify high-risk groups for seroma formation in routine clinical work and take active preventive measures.

#### Adjuvant RT

There are literatures on postoperative adjuvant RT, showing that postoperative adjuvant RT can improve the overall survival (OS) and disease-specific survival of patients after receiving local surgery [36]. Khanfir et al. retrospectively analyzed 61 breast ACC patients undergoing BCS. The result indicated that 5-year local area control rate of patients with adjuvant RT was higher than those without RT. The author suggested that BCS should be the preferred treatment for patients with breast ACC and adjuvant RT could bring more benefits to patients [37].

#### Adjuvant CT

At present, there are still controversies about adjuvant CT after operation and no consensus is reached. Arpino G et al. suggested that postoperative adjuvant CT did not improve disease free survival or OS in breast cancer patients [3]. Treitl et al. investigated six patients with breast ACC, and none of them were found accompanied with lymph node metastasis. The researcher assumed that patients with breast ACC did not need adjuvant CT after operation [38]. Coincidentally, there is another study in which only 11.3% of all patients receive adjuvant CT after surgery [24]. However, for patients with axillary lymph node metastasis, some experts claim that systematic adjuvant CT is necessary and for patients with high-grade or large tumor with diameter larger than 3 cm, adjuvant CT should be considered [10]. In all, the relationship between postoperative adjuvant CT and prognosis of ACC patients needs to be further explored.

#### Adjuvant endocrine therapy

ACC of breast is often regarded as a subtype of TNBC. Therefore, endocrine therapy is unnecessary. Yigit retrospectively reviewed seven patients diagnosed with breast ACC. IHC showed that expression of progesterone receptor (PR) and HER2 was absent in all patients. Only one patient showed weak positive expression of estrogen receptor (ER). Besides, six of them showed positive expression of androgen receptor. Therefore, the author presented that hormone therapy could be applied in androgen receptor positive patients in the future [39]. However, more in-depth studies are needed to confirm this viewpoint. Vranic et al. tested IHC from eleven breast cancer patients and found that eight patients expressed ER-α36 while no patients expressed ER-α66, PR or HER2. This study indicated that ER-α36 was a novel subtype of ER-α66 and was overexpressed in breast ACC frequently [40]. ER-α36 may act as a new target for endocrine therapy in the future.

## Conclusions

Breast ACC differs from traditional TNBC. It is characterized by slower clinical process and lower invasiveness. It is necessary to diagnosis breast ACC clearly. Standardized treatment is our objective to avoid the physical and psychological harm for patients caused by overtreatment or undertreatment. At present, BCS and mastectomy are widely used clinically. Generally, ALND is evitable. More large-scale studies are needed to confirm whether CT is beneficial for patients. Although axillary lymph node metastasis is rare, the possibility of distant metastasis should not be ignored. Regular review and long-term follow-up for patients are absolutely necessary.

## Data Availability

All data generated or analysed during this study are included in this published article.

## Abbreviations

ACC: Adenoid cystic carcinoma
TNBC: Triple-negative breast cancer
IHC: Immunohistochemistry
HER2: Human epidermal growth factor receptor 2
MRM: Modified radical mastectomy
BCS: Breast conservation surgery
SLNB: Sentinel lymph node biopsy
CT: Chemotherapy
RT: Radiotherapy
NFIB: Nuclear factor IB
SBACC: Solid breast adenoid cystic carcinoma
ER: Estrogen receptor
PR: Progesterone receptor
ALND: Axillary lymph node dissection
OS: Overall survival
UOQ: Upper outer quadrant
UIQ: Upper inner quadrant
LIQ: Lower inner quadrant
HT: Hormone therapy
